# Emerging concepts in intestinal immune control of obesity-related metabolic disease

**DOI:** 10.1038/s41467-021-22727-7

**Published:** 2021-05-10

**Authors:** Saad Khan, Helen Luck, Shawn Winer, Daniel A. Winer

**Affiliations:** 1 Department of Immunology, University of Toronto, Toronto, ON Canada; 2 Division of Cellular & Molecular Biology, Diabetes Research Group, Toronto General Hospital Research Institute (TGHRI), University Health Network, Toronto, ON Canada; 3 Department of Laboratory Medicine and Pathobiology, University of Toronto, Toronto, ON Canada; 4 Department of Laboratory Medicine, St. Michael’s Hospital, Toronto, ON Canada; 5 Department of Pathology, University Health Network, Toronto, ON Canada; 6 Buck Institute for Research on Aging, Novato, CA USA

**Keywords:** Mucosal immunology, Clinical microbiology, Metabolic syndrome, Obesity

## Abstract

The intestinal immune system is an important modulator of glucose homeostasis and obesity-associated insulin resistance. Dietary factors, the intestinal microbiota and their metabolites shape intestinal immunity during obesity. The intestinal immune system in turn affects processes such as intestinal permeability, immune cell trafficking, and intestinal hormone availability, impacting systemic insulin resistance. Understanding these pathways might identify mechanisms underlying treatments for insulin resistance, such as metformin and bariatric surgery, or aid in developing new therapies and vaccination approaches. Here, we highlight evolving concepts centered on intestinal immunity, diet, and the microbiota to provide a working model of obesity-related metabolic disease.

## Abstract (web summary)

At the centre of intestinal health and the fight against obesity and metabolic syndrome is the intestinal microbiota and its interaction with our immune systems. Here the authors Review the current understanding of how these systems interact and how we can capitalize on recent advances to provide better therapeutic options.

## Introduction

Human obesity is an excessive accumulation of adipose tissue (adiposity) clinically defined by the World Health Organization as constituting a body mass index (BMI) > 30 kg/m^2^ ^1^. Obesity can be considered a growing epidemic that is associated with complications, including hyperglycemia, insulin resistance, and dyslipidemia, collectively referred to as metabolic syndrome^1^. Metabolic syndrome is a state of low-grade inflammation that supports the development of chronic diseases such as type 2 diabetes (T2D) mellitus, non-alcoholic fatty liver disease (NAFLD), and cardiovascular disease (CVD). In mice, aspects of metabolic syndrome are often modeled using high-fat diet feeding, in which 40~60% of caloric intake is acquired from dietary fat, resulting in the development of diet-induced obesity and associated metabolic defects, such as insulin resistance, and accompanying low-grade inflammation^2^.

Identifying the root cause of this low-grade inflammation is a developing avenue of research. Both adaptive and innate immune cell-mediated inflammation can occur in metabolic tissues, such as visceral adipose tissue (VAT) and the liver of individuals who are obese^3,4^. The intestines also display alterations in immune composition during obesity and function as a focal point of crosstalk between intestinal barrier function and intestinal microbiota^5^. Obesity is not only associated with a change in the composition of intestinal immune cells but also immune cell-secreted factors that shape the intestinal microbial composition, such as bacteria-specific antibodies^6,7^. In turn, a dysbiotic microbiome is an important factor linking the immune system with obesity-associated disease outcomes^8^. Moreover, a role for intestinal immune cells in controlling intestinal hormone bioavailability has been uncovered, expanding the physiological function of the immune system in endocrine regulation^9^. These exciting findings have pushed the field forward and highlight many current questions. Specifically, how do the intestinal immune system and microbiota regulate one another? What consequences does this crosstalk have for the development of obesity-associated metabolic disease? Do existing therapies for insulin resistance target these axes, and can we use our understanding of intestinal immune microbial crosstalk to develop new therapeutic strategies? Finally, how can we rationalize the intestinal immune system as the major focal point for the induction of metabolic syndrome?

In this Review, we discuss some of the most recent advances in intestinal immunometabolism and microbiology in the context of obesity and associated metabolic disease. We also discuss gaps in knowledge and propose avenues of research that might yield new insight into immune cell-microbiota crosstalk and the development of therapeutic modalities.

## A pathogenic intestinal immune compartment

**Fig. 1:**
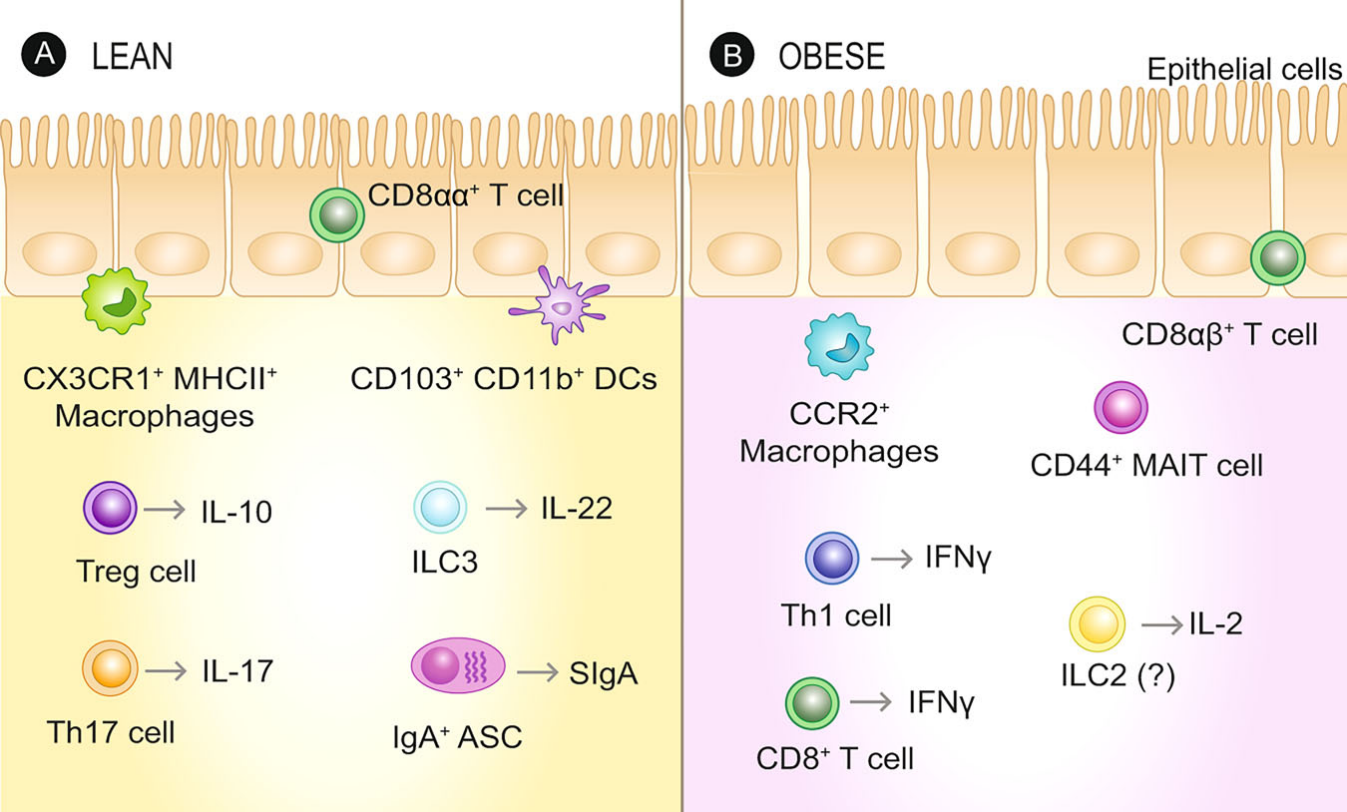
Obesity alters the intestinal immune compartment. **A** Under lean homeostatic conditions, the intestinal immune environment is dominated by tolerogenic and mucosal barrier maintaining immune cells. These cells include interleukin (IL)-10 producing regulatory T (Treg) cells, IL-22 producing group 3 innate lymphoid cells (ILC3s), and IL-17 secreting protective T helper (Th) 17 cells. Furthermore, under lean conditions, IgA^+^ antibody-secreting cells (ASCs) are abundant within the lamina propria and produce secretory IgA (SIgA), which interface with the intestinal bacteria. The lean intestinal environment also consists of tolerogenic CX3CR1^+^ MHC-II^+^ macrophages and CD103+ CD11b+ dendritic cells (DCs), which have been linked to protective Th17, ILC3, and IgA responses within the intestines. **B** During diet-induced obesity in mice, there is a shift in the inflammatory potential of the intestinal immune environment, leading to increased numbers of lamina propria Th1 and CD8^+^ T cells, CD44^+^ MAIT cells, intestinal homing CCR2^+^ macrophages, and intestinal intra-epithelial CD8αβ^+^ T cells, as well as a decrease in the number of the aforementioned tolerogenic cell types. Additionally, small intestine ILC2s have been shown to promote obesity via an IL-2 feedback system, and further work may delineate the basis of this axis. In human subjects with obesity, there is an increase in intestinal CD8αβ^+^ T cells. These changes result in an inflammatory environment that is linked with intestinal dysfunction, facilitating dysregulated glucose homeostasis during diet-induced obesity.

Obesity in mice and humans is associated with alterations in the composition of the intestinal immune system (Fig. 1). These shifts are observed in both the adaptive and innate arms of the immune system, which facilitates the emergence of a broadly pro-inflammatory intestinal landscape capable of driving the development of obesity-associated metabolic disease. Here we present an up-to-date summary of obesity-associated alterations to the intestinal immune compartment, focusing on the most recent findings, and provide direction for future research. We further highlight important mechanisms controlling dysfunctional intestinal immune cells that contribute to the development and progression of obesity-associated metabolic disease.

### Aberrant pro-inflammatory shifts in intestinal immune cells during obesity

Cells of the adaptive immune system are critical for an effective response against infectious agents and for the induction of immune memory. In the intestine, these cells are central for mutualism between the host and the intestinal microbiota^10^. Obesity is associated with important changes to the adaptive immune compartment in the intestines, which results in aberrant inflammatory skewing. In particular, in the intestinal lamina propria, the number of pathogenic interferon-γ (IFN-γ)-producing T helper 1 (Th1) and CD8αβ^+^ T cells is increased, and the number of protective interleukin 17 (IL-17)-producing Th17 cells and IL-10-secreting regulatory T (Treg) cells is decresaed^2,11–13^ In humans, increased numbers of CD8αβ^+^ T cells are also detected in the intraepithelial fraction^13^. T cell classification within the intestine can be further narrowed, and more work is needed to clarify the function of these specific immune cell subsets in obesity. For example, within the intestinal niche, two broad subsets of Treg cells can be separated by the expression of Helios, GATA3, and RORγt^14,15^. Helios^+^ GATA3^+^ Treg cells are of thymic origin, whereas Helios^−^ RORγt^+^ Treg cells differentiate locally in response to the microbiota, are dependent on the transcription factor c-Maf, and are critical in limiting the expansion of Th17 cells^15,16^. Indeed, T cell subsets in the intestine reflect dynamic responses shaped by the microbiota^17^. Two distinct subsets of Th17 cells that differ in their metabolic machinery, as well as their inflammatory output, are also present within the intestines^18^. Homeostatic Th17 cells have a muted inflammatory profile and can be involved in barrier protection, whereas pathogenic Th17 cells secrete large amounts of IFNγ^18^. These characteristics make it important to discriminate Th17 populations by their cytokine secretion profile, as it is possible that obesity is associated with an overall targeted reduction in homeostatic Th17 cells.

In addition to T cells, intestinal B cells and terminally differentiated antibody-secreting cells (ASCs) have been shown to regulate obesity-associated metabolic disease. Diet-induced obesity is associated with a reduction in intestinal IgA^+^ ASCs and B cells, which results in a decrease in colonic secretory IgA and contributes to the development of insulin resistance^6^. Additionally, some ASC subsets, including intestinal IgA^+^ ASCs, can secrete anti-inflammatory cytokines, such as IL-10, which can dampen local tissue inflammation; however, the effect these cells have in the context of obesity is unclear^19^. Although B1 and B2 B cells have been examined extensively in the context of regulating adipose tissue inflammation and insulin resistance, little is known about the function of these cell subsets in the intestines in the context of systemic glucose homeostasis^20,21^.

Under steady-state, cells of the innate immune system are at the forefront in combating pathogens, and although these cells have limited memory responses they are crucial for effective activation and control of adaptive immune cells^22^. Obesity is associated with several disruptions in the innate intestinal immune compartment, which is subsequently linked with the development of insulin resistance. Notably, the frequency of IL-22 and IL-17-secreting intestinal group 3 innate lymphoid cells (ILC3s) in mice is reduced, an effect that is crucial for maintaining barrier integrity of the intestinal epithelium^2^. A pathogenic effect of small intestinal group 2 innate lymphoid cells (ILC2s) has also been shown, as mice deficient in these cells are protected from metabolic syndrome^23^, and adoptively transferring these cells into ILC2-deficient mice can recapitulate parameters of metabolic disease^23^. Interestingly, during diet-induced obesity in mice, small intestinal ILC2s have been shown to be functionally distinct from adipose tissue ILC2s, as they produce more IL-2^23^, which further preferentially maintains intestinal ILC2s^23^. However, ILC2s in the intestine is also a prominent source of IL-10, and as such, it is unclear if all of these cells become pathogenic during diet-induced obesity^24^. In humans, the number of intestinal ILCs has been shown to negatively correlate with BMI, but the functional consequences of this effect are unclear^25^.

Monocytes, dendritic cells, and macrophages have been implicated in regulating metabolic dysfunction. A CCL2-dependent influx of pro-inflammatory CCR2^+^ macrophages into the intestines in high fat diet-fed mice is associated with low-grade inflammation and metabolic dysfunction^26^. Accordingly, the number and activity of CX3CR1^+^ lamina propria macrophages decreases in high-fat diet-fed mice^6,12^. Although a reduction in the number of CX3CR1^+^ macrophages might limit induction of protective Th17 cells and IgA class switch recombination, whether or not an influx of CCR2^+^ macrophages is directly associated with the loss in CX3CR1 macrophages has not been shown^6,12,26^. Intestinal monocyte turnover during obesity could be further studied using Tim4 and CD4 as markers, as under steady-state Tim4^+^ CD4^+^ macrophages are maintained locally, independent of monocyte contribution, whereas Tim4^−^ CD4^+^ and Tim4^−^ CD4^−^ are differentially replenished by infiltrating monocytes^27^. Whether diet-induced obesity is associated with altered macrophage heterogeneity and if such alterations would correlate with obesity-associated intestinal dysfunction is presently unclear. The intestines also maintain a heterogeneous population of dendritic cells that can be categorized into conventional dendritic cells (cDCs) and plasmacytoid dendritic cells (pDCs). cDCs can be further subdivided into CD103^+^ CD11b^−^ cDC1s, CD103^−^ CD11b^+^ cDC2s, and C103^+^ CD11b^+^ intestine-specific cDCs^28^. Although high-fat diet feeding in mice can induce shifts within the cDC compartment, thereby decreasing the abundance of pro-IgA intestinal-specific CD103^+^ CD11b^+^ cDCs, thorough functional phenotyping of intestinal DCs during obesity is required^6^.

Further studies are also needed to characterize changes in intestinal innate-like T cells during obesity-associated metabolic disease. Lipid presentation to NKT cells via CD1d is potentially important for the regulation of intestinal homeostasis^29^, but whether this homeostatic function is altered during obesity has not been examined. Mucosal-associated invariant T (MAIT) cells are innate-like T cells that recognize bacterial metabolites presented by MR1 and are typically dependent upon exposure to defined microbial communities rich in riboflavin-synthesizing bacteria for development^30^. MAIT cells produce beneficial IL-17 and can regulate barrier function by attenuating pathogenic intestinal T cell responses^31^. Obesity in mice is associated with a reduction in the number of MAIT cells in the small intestine, yet those cells that persist have an activated CD44^+^ phenotype, which contributes to intestinal inflammation and the development of insulin resistance^32^. Factors that promote this shift in the inflammatory status of MAIT cells during obesity are not identified and require further examination.

### Mechanisms for intestinal immune cell promotion of metabolic disease

**Fig. 2:**
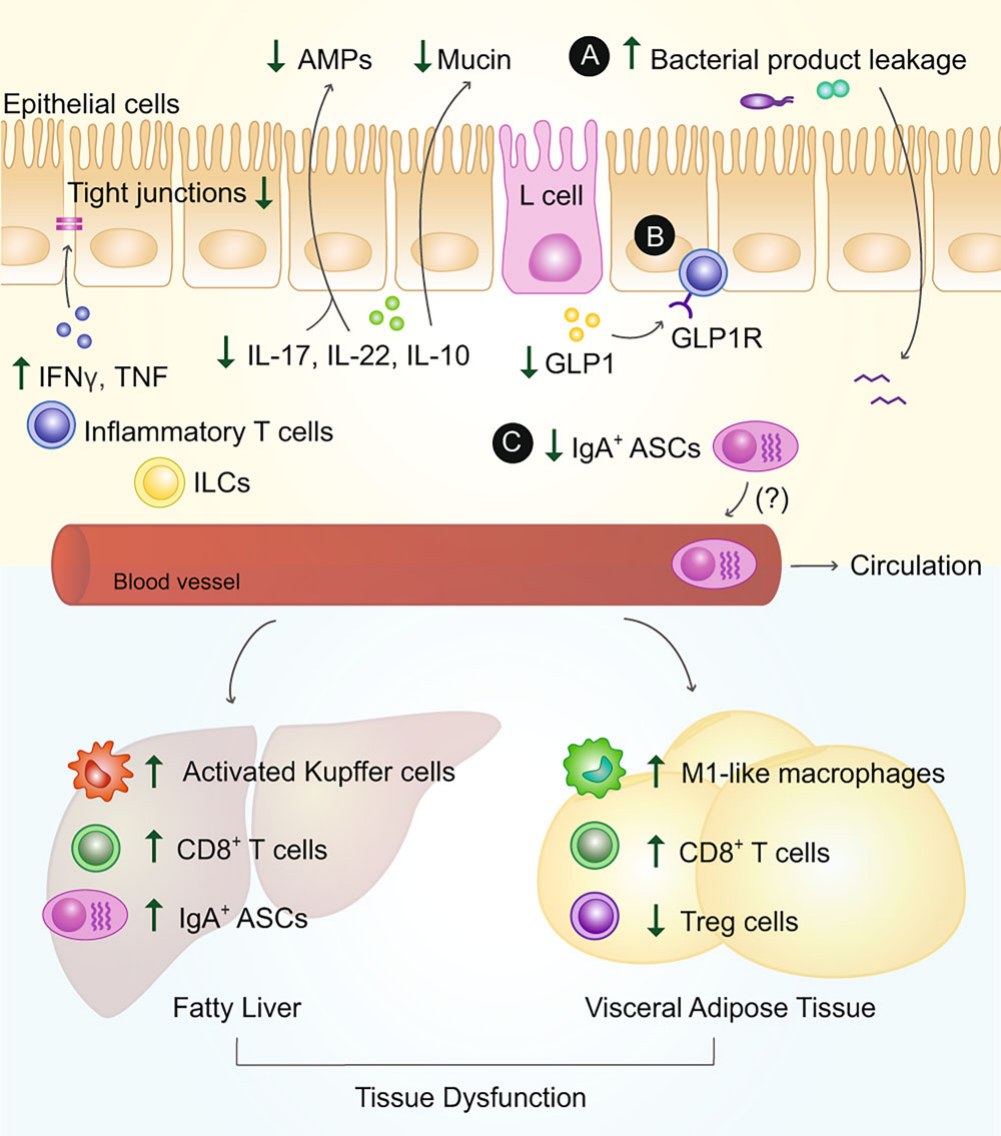
Mechanisms of intestinal immune cell-driven metabolic dysfunction. During obesity, intestinal immune cells contribute to insulin resistance and/or glucose dysregulation, via at least three potential mechanisms. **A** Changes in immune compartments in the presence of an obesogenic diet promote leakage of bacterial products. This intestinal permeability is facilitated through local changes in cytokines, such as increases in pro-inflammatory cytokines interferon (IFN)γ and tumor-necrosis factor (TNF), coupled with a loss in anti-inflammatory and barrier protective cytokines interleukin (IL)-17, IL-22, and IL-10, anti-microbial peptides (AMPs) and epithelial mucin. Changes in such factors also contribute to the degradation of epithelial tight junction proteins. Increased leakage of bacterial products can drain into metabolic tissues, such as the visceral adipose tissue (VAT) and liver, where they further stimulate local immune cells, such as hepatic Kupffer cells and VAT M1-like macrophages, further enhancing their pro-inflammatory profile and leading to systemic insulin resistance. **B** Intestinal lymphocytes can potentially sequester gastrointestinal hormones such as glucagon-like peptide (GLP)1, via their GLP1 receptor (GLP1R), thereby limiting its bioavailability and further contributing to metabolic dysfunction. **C** Finally, evidence indicates that some intestinal immune cells, such as anti-inflammatory IgA^+^ antibody-secreting cells (ASCs), can potentially migrate to distant inflamed sites in the body, resulting in the reduced intestinal presence of ASC products such as IL-10 and IgA.

How changes in the intestinal immune composition during obesity affect systemic parameters of metabolic disease is an important question. In particular, is there a role for intestinal immune cells in contributing to the development of chronic low-grade inflammation at metabolic sites that facilitate insulin resistance, or in the control of hormones that regulate insulin production? Here, we describe a number of important intestinal immune cell-mediated mechanisms that facilitate the development of obesity-associated metabolic tissue inflammation, insulin resistance, intestinal hormone bioavailability, and glucose dysregulation (Fig. 2).

Our understanding of the mechanisms by which an altered intestinal immune landscape mediates metabolic dysfunction has previously focused on perturbation of intestinal barrier integrity. During obesity, the pro-inflammatory skewing of immune cells favors the release of cytokines such as TNF and IFNγ, coupled with a reduction in IL-10, IL-17, and IL-22, resulting in reduced expression of epithelial tight junction proteins, mucin, and antimicrobial proteins such as RegIIIγ^2,33^. This barrier dysfunction has long been hypothesized to be an important driver of metabolic syndrome, as increased levels of bacterial products promote inflammation in important metabolic tissues leading to insulin resistance^34^. For example, NAFLD and progression to non-alcoholic steatohepatitis (NASH) are, in part, driven by bacterial products (including unmethylated CpG DNA) entering the liver, ultimately activating intra-hepatic Kupffer cells and CD8^+^ T cells resulting in inflammation, hepatocyte injury and death, insulin resistance with associated increased hepatic gluconeogenesis, and fibrosis^35,36^. In accordance with these collective findings, treating obese mice with intestinal anti-inflammatory agents, such as 5-aminosalicylic acid, can reduce intestinal permeability and endotoxemia and improve the overall metabolic phenotype^2^.

Intestinal immune cells further contribute to metabolic disease by regulating the bioavailability of gastrointestinal hormones, which subsequently control blood glucose levels. Under homeostatic conditions, intestinal intraepithelial lymphocytes express the glucagon-like peptide 1 receptor (GLP1R), which, upon signaling, reduces the production of pro-inflammatory cytokines^37^. Glucagon-like peptide 1 (GLP1) is an incretin that enhances the secretion of insulin to reduce blood glucose levels; during obesity, intestinal epithelial lymphocytes can sequester GLP1 to limit its bioavailability^9^. Obesity is also associated with a reduction in GLP1-secreting enteroendocrine L cells^9^, which further contributes to reduced GLP1 levels. In the pro-inflammatory setting of obesity, activated intestinal intraepithelial lymphocytes might also upregulate the expression of molecules (such as dipeptidyl peptidase 4 (DPP4)) that degrade GLP1 as another means to reduce GLP1 bioactivity during obesity^38^. Chronic exposure of L-cells to TNF can hamper GLP1 production in vitro^39^, but whether intestinal intraepithelial lymphocyte-derived pro-inflammatory cytokines directly contribute to a loss of L cells in vivo, or potentially modulate levels of other gastrointestinal hormones warrants further investigation. The reciprocal effect of intestinal hormones on local immune cells would also be interesting to explore. For example, cholecystokinin, serotonin, and ghrelin might regulate T cell activation and differentiation of naïve T cells to Th17 and Treg fates^40–42^. Intestinal-derived hormones, as well as neuropeptides, can regulate levels of intestinal IgA and myeloid cell activity, which have altered phenotypes and functionality during obesity^43,44^. The crosstalk between intestinal endocrine cells and intestinal immune cells is an exciting future direction of research that might uncover mechanisms of intestinal immune cell pathogenicity.

Finally, evidence indicates that some intestinal immune cells can migrate to inflamed metabolic sites^45^. Under homeostatic conditions, the liver hosts a population of IgA^+^ ASCs of intestinal origin, which is further expanded during liver disease^46^. Interestingly, these intestinal ASCs can also access the systemic circulation and migrate to inflamed sites, such as the brain in a mouse model of multiple sclerosis, where they can be immunosuppressive by secreting IL-10^19^. Similarly, anti-inflammatory effects of hepatic IgA^+^ ASCs on CD8^+^ T cells are associated with the progression of hepatocellular carcinoma^47^. During obesity-associated metabolic disease, intestinal IgA^+^ ASCs, potentially targeting commensal microorganisms or self-antigens (e.g., through antigenic mimicry), might traffic to inflamed metabolic tissues in an attempt to mitigate inflammation. Such an idea might explain the reduction in their presence in the intestine during diet-induced obesity^6^. However, this migration away from the intestines would come at the expense of the intestinal barrier, as a lack of these cells could lead to increased intestinal inflammation and intestinal permeability^6^. Accordingly, accumulation of MAIT cells in the liver during metabolic syndrome has also been documented^48^, but whether this increase is linked to the loss of these cells due to migration from the small intestine is not clear^32^.

## Expansion of opportunistic microbes

Although intestinal immune cells can contribute to metabolic disease by promoting intestinal barrier dysfunction, altering the bioavailability of gastrointestinal hormones, and migrating to distant sites during disease, these mechanisms are affected by other critical factors, such as crosstalk between obesity-related microbiota and their microbial metabolites. Here, we review alterations to the intestinal microbiota and their metabolome that are linked with the development of obesity-associated metabolic disease.

### Bacterial dysbiosis is a hallmark of metabolic syndrome

Obesity is associated with an altered intestinal microbial composition, a state termed dysbiosis. During obesity-related dysbiosis, the intestinal bacterial diversity is reduced and intestinal microbes have enhanced capacity for nutrient harvesting from the diet^49^. These features have been linked to obesity-associated clinical consequences such as insulin resistance and dyslipidemia^49,50^. Fecal matter transplant from obese mice and obese human donors into germ-free mice can recapitulate parameters of obesity-associated metabolic dysfunction^49,51^, providing evidence that intestinal microbes can be pathogenic. Although much work on obesity-associated microbial dysbiosis has explored differences in the ratio of Bacteroidetes to Firmicutes, the metabolic syndrome also seems to be associated with enrichment of Proteobacteria, a phylum that is a potential diagnostic signature of intestinal dysbiosis^49,52^. For example, an abundance of Proteobacteria correlates with the severity of fibrosis in patients with NAFLD^53^, and inoculating mice with either the Proteobacteria *Enterobacter cloacae*^54^ or *Bilophila wadsworthia*^55^ promotes metabolic disease in mice. Such findings implicate Proteobacteria as being involved in the pathogenesis of metabolic syndrome, but the mechanisms that promote the expansion of this opportunistic taxa require further attention. Dietary triggers or metabolites might promote growth, activity and establishment at the expense of commensal bacteria. Alternatively, expansion of Proteobacteria numbers might be a consequence of intestinal inflammation, as can occur in dextran sulfate sodium (DSS) induced colitis in mice^56^, or due to a loss of neutralizing IgA antibodies^6,7^. Identifying factors that promote the abundance of opportunistic microbes at the cost of mutualistic commensals should provide a better framework to understand environmental interactions between the host and its microbiome in the context of diet-induced obesity.

Broad interest also exists in replenishing intestinal levels of beneficial microbes, for example with *Akkermansia muciniphila* of the Verrucomicrobia phylum. Although there is limited evidence for an effect of obesity on the abundance of *Akkermansia muciniphilia*, oral administration of live and pasteurized microbes, via gavage (mice) and medicinal packages (humans), can improve outcomes associated with metabolic syndrome and liver dysfunction^57,58^. As the pasteurized form of this bacterium was also effective, it raises the possibility that exposure to high levels of a commensal antigen might have tolerizing effects on intestinal immunity^57,58^.

### Bacterial metabolites affect metabolic disease

Obesity-associated microbial dysbiosis accompanies a change in the presence of important bacterial metabolites, which are now regarded as a means to mediate bacterial effects on the host^59^. For example, a Western diet is associated with an alteration in microbial fermentation of indigestible carbohydrates, such as dietary fibers, that would typically produce metabolites such as short chain fatty acids (SCFAs) that can inhibit lipogenesis and promote lipid oxidation^49,60,61^. Fermentation of dietary fibers also produces succinate, which can directly stimulate intestinal gluconeogenesis to improve glucose homeostasis^62^. Other metabolites that have been identified to mediate beneficial effects and ameliorate obesity-associated metabolic dysfunction include tryptophan-based compounds which signal via the aryl hydrocarbon receptor^63^ and secondary bile acids that can function through the farnesoid X receptor (FXR)^64,65^. As an example, bile acids produced in the liver are metabolized by the intestinal microbiome to yield bioactive secondary bile acids^66^. These bile acids have signaling properties, and interestingly post-bariatric surgery insulin sensitization in individuals with obesity can be mediated by alterations in microbial bile acid metabolite FXR signaling^67^. How these beneficial microbial metabolites are affected during obesity and how they can be used to improve obesity-related outcomes, is an exciting avenue of future research.

During obesity, there is an upregulation of several pathogenic metabolites, such as trimethylamine, which is enzymatically converted to trimethylalamine N-oxide, an organic compound that is associated with cardiometabolic disease^68^. Similarly, microbial-produced imidazole propionate, a histidine derivative, has been shown to be elevated in the portal vein of patients with T2D and is capable of impairing insulin signaling at the level of insulin receptor substrate via activation of the mechanistic target of rapamycin complex 1 (mTORC1)^69^. Intestinal microbe-derived metabolites that affect host physiology have been reviewed elsewhere^59^. This list will continue to expand in the coming years and should yield further insights into the regulation of metabolic disease.

### Pathogenicity of obesogenic intestinal microbes extends to metabolic sites

Evidence indicates that an obesity-associated intestinal microbiota alters important organ-specific metabolic processes in the adipose tissue and liver that ultimately affect systemic insulin resistance and glucose dysregulation. Cells within the adipose tissue can be converted into thermogenic energy dissipating brown-like adipocytes in a process termed beiging, which can be mediated by adipose tissue ILC2s and eosinophils, and has strong insulin-sensitizing effects, yet is compromised during obesity^70,71^. Versatility in the intestinal microbiome contributes to thermogenesis, as a cold-induced microbiome can promote beiging and insulin sensitization^72^. Additionally, depletion of the homeostatic commensal microbiome can impair thermogenesis, and amelioration of an obesity-associated microbiome or supplementation with SCFA butyrate can restore thermogenic processes and improve obesity-associated glucose tolerance and insulin sensitivity^73,74^. Similarly, the intestinal microbiome is crucial to the regulation of hepatic metabolic processes, such as gluconeogenesis and insulin sensitivity, in a manner that is dependent on intestinal metabolite secretion^69,75^. As such, features of the crosstalk between the intestines, liver and VAT can alter metabolic disease parameters during obesity and are an important area of research that explores the function of microbial metabolites beyond the intestinal tract. Pioneering work has demonstrated that metabolic tissues such as the liver and VAT from individuals who are morbidly obese have unique inter-organ microbial signatures and that tissue-specific taxa differ between individuals with and without T2D^76^. Importantly, Proteobacteria are highlighted as a predominant phylum within the liver during NAFLD that is associated with lobular and portal inflammation^77^. Similarly, Proteobacteria is a predominant phylum in the adipose tissue of patients with T2D and can induce local adipose-specific pro-inflammatory responses^78^. Furthermore, heterogeneity in bacterial composition and abundance can occur between adipose tissue depots, which is further associated with markers of inflammation and insulin resistance^78^. Interestingly, in patients with T2D, the mesenteric adipose tissue surrounding the intestines has the greatest diversity and number of bacterial operational taxonomical units, suggesting it might function as an initial ‘gatekeeper’ between the intestines and systemic exposure to bacteria during obesity^76,78^. Cumulatively, these findings suggest that translocation of intact opportunistic microbes from the intestines to metabolic sites might directly alter local tissue immune landscapes, thereby promoting the development of glucose intolerance and insulin resistance in individuals who are obese.

## The microbiome–immune cell axis

**Fig. 3:**
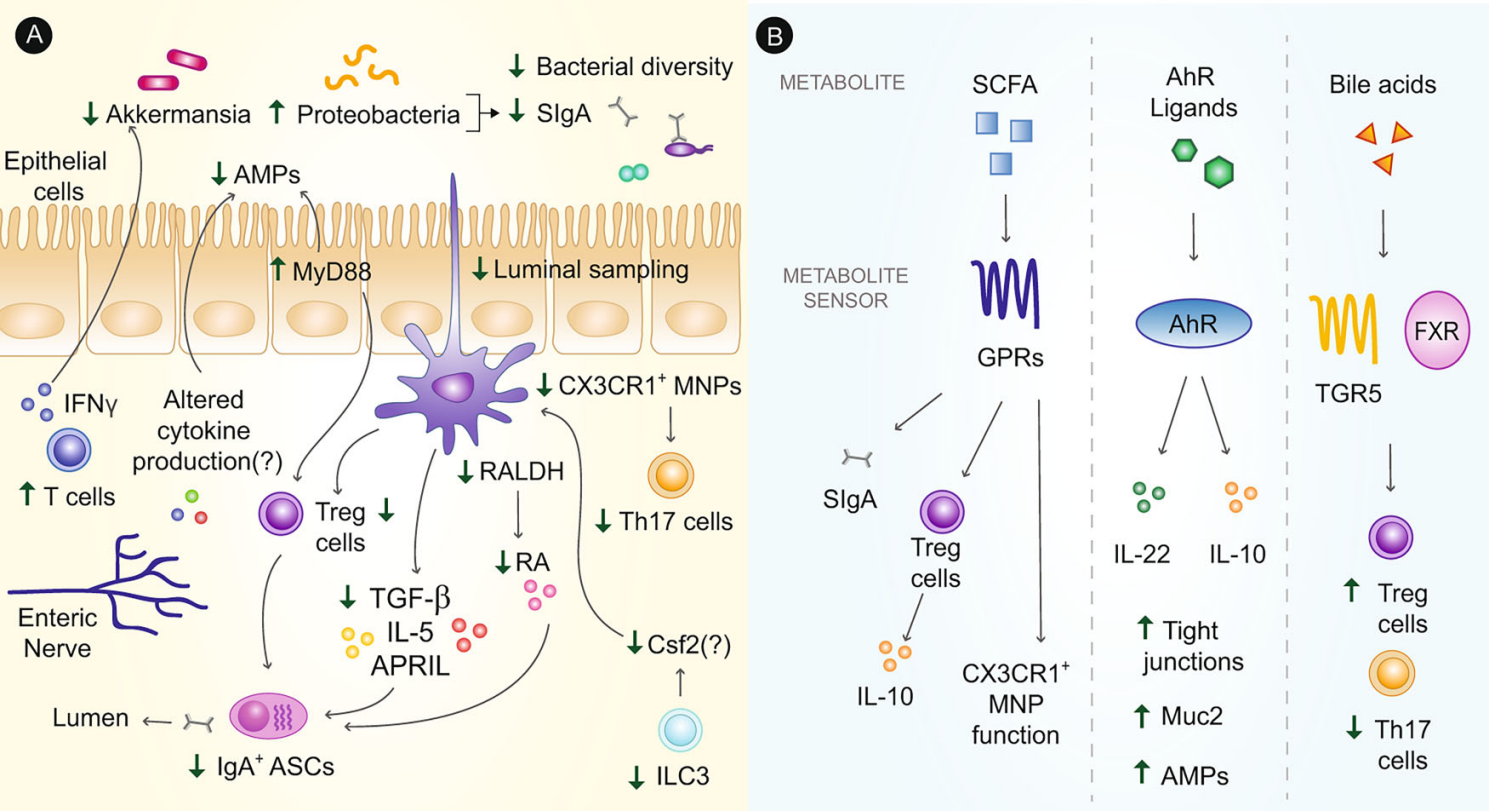
Immune-microbiota crosstalk regulates metabolic disease. **A** During obesity, altered intestinal immune cell function is tightly linked with intestinal dysbiosis and a loss in bacterial diversity. Mononuclear phagocytes (MNPs; macrophages and dendritic cells - CX3CR1+ macrophages shown in figure as an example) have an altered ability to produce factors necessary for IgA class switching, such as transforming growth factor-beta (TGF-β), interleukin-5 (IL-5), retinoic acid (RA) via retinaldehyde dehydrogenase (RALDH) enzymes, and a proliferation-inducing ligand (APRIL). This reduction would directly affect the amount and quality of secretory IgA (SIgA) present to bind bacteria, enabling the expansion of opportunistic and pathogenic taxa, such as Proteobacteria, and promoting obesity-associated dysbiosis. Defects in MNP functionality is further affiliated with reduced secretion of factors linked to IgA antibody-secreting cell (ASC) function, and reduced ability to induce T helper 17 (Th17) and regulatory T (Treg) cells. These defects potentially can be further affected by a decreased colony-stimulating factor (Csf)-2 production, as obesity induces a reduction in intestinal group 3 innate lymphoid cells (ILC3s), but this remains to be examined. Simultaneously, pro-inflammatory signaling in intestinal epithelial cells, T cells, and potentially enteric neurons, alters the production of anti-microbial peptides (AMPs), or can directly hinder the beneficial function of beneficial taxa like Akkermansia, contributing to microbial dysbiosis. **B** The microbiota in turn exert control on immune cells through secretion of metabolites, which are affected by diet-induced obesity. For example, short-chain fatty acids (SCFAs) function through their receptors, such as G-protein coupled receptors (GPRs), to promote levels of intestinal secretory IgA, boost Treg cell responses and promote CX3CR1^+^ MNP function. Aryl hydrocarbon receptor (AhR) ligands have been associated with increased interleukin (IL)-22 and IL-10 cytokine production, AMP production, and the promotion of epithelial layer mucus and tight junction proteins. Via receptors such as the farnesoid x receptor (FXR) and G-protein coupled bile acid receptor (TGR5), bile acid metabolites might also regulate the intestinal immune landscape through the promotion of Treg cells. These processes are potentially reduced or altered with a Western diet, contributing to inflammatory immunological changes in the intestines during obesity.

The crosstalk between intestinal immune cells and microbial species is essential for immune function. Alterations in this crosstalk have been linked with the progression of metabolic disease and are an important emerging area of obesity-related insulin resistance research (Fig. 3), which we discuss in this section.

### Intestinal immune control of the microbiota during obesity

Under homeostatic conditions, IgA is secreted across the intestinal epithelium in its dimeric form as secretory IgA, which binds to bacteria and their products to regulate microbiota composition and reduce microbial penetration across the intestinal barrier^79^. Reduced levels of secretory IgA in the stool during diet-induced obesity in mice leads to dysbiosis, characterized by increases in the abundance of Proteobacteria and decreases in some Clostridia, coupled with increased intestinal inflammation and reduced intestinal barrier integrity^6^. Concurrently, during diet-induced obesity in mice, IgA is bound to bacteria at a higher affinity, confirming its ability to bind to pathogenic species^6,79^. IgA binding can potentially also alter the functionality of obesity-associated microbes, such as global gene expression patterns, affecting their motility and local occupancy within the intestinal environment^79^. Although the antibody isotype IgM can partially compensate for a loss in IgA in some circumstances, humans with selective IgA deficiency (a form of primary immunodeficiency disease) have an altered intestinal microbiota despite secreting compensatory IgM^80,81^. Nonetheless, IgM is polyreactive to many bacterial species and whether intestinal production and functionality of this antibody is also altered during metabolic disease has not been examined.

As IgA can be generated via T cell-dependent and T cell-independent mechanisms that control the polyreactivity and affinity of the antibody and which microbes they coat, identifying which pathway is defective during obesity is an important consideration^79^. T cell-dependent high-affinity IgA production relies on the use of T follicular helper (Tfh) cells, which have been linked with the production of IgA in a diet-induced model of obesity^7^. Defective Tfh cell function and IgA coating result in a loss of Clostridia and expansion of the Proteobacteria *Desulfovibrio* population, facilitating dysfunctional lipid metabolism, findings that are in line with studies of IgA-deficient mice^6,7^.

Although we have a reasonable understanding of defective T cell-dependent IgA production and function during obesity, polyreactive T cell independent IgA requires further study. Under homeostasis, microbial signals induce intestinal ILC3 populations to produce colony-stimulating factor 2 (Csf2), which maintains local mononuclear phagocytes and their ability to express the retinoic acid-generating enzyme retinaldehyde dehydrogenase and secrete TGFβ^82^. Furthermore, mononuclear phagocytes respond to cues from microbes to modulate their function. This includes orchestrating the production of tertiary lymphoid structures and resultant high-affinity IgA antibodies^83^, as well as T cell-derived IL-17 and IL-22 responses^84^. These cellular factors, coupled with secreted factors including IL-5, TGFβ, and a proliferation-inducing ligand (APRIL), support the presence of intestinal IgA and are reduced within the intestinal lamina propria during diet-induced obesity^6^. Therefore, it might be possible that defective ILC3 and mononuclear phagocyte sensing of the microbiota functions as a mechanism for reduced IgA responses, which in turn regulate the intestinal microbiota during obesity.

Intestinal epithelial cells also confer innate immunity against microbes via their secretion of mucin, anti-microbial peptides, and endocannabinoids^85,86^. During chronic obesity, aberrant intestinal epithelial cell MyD88 signaling in mice fed a high-fat diet has been linked with the development of obesity-associated insulin resistance, by restricting secretion of endocannabinoids and antimicrobial peptides, and promoting a pro-inflammatory intestinal environment^86^. Adherence of microbes to intestinal epithelial cells also induces tolerant intestinal Th17 responses^87^. Future research should study whether obesity interferes with commensal adhesion and associated induction of tolerogenic cytokine responses^87^.

IFNγ also contributes to microbial control during metabolic disease. IFNγ reduces levels of *A. muciniphila* by modulating the expression of intestinal *Irgm1*^88^. As diet-induced obesity is associated with increasing numbers of intestinal IFNγ-expressing T cells, this IFNγ-*Irgm1* axis probably constitutes another important means by which immune cells alter bacterial taxa to promote metabolic disease.

Finally, enteric neurons are a source of cytokines that can counter bacterial infections, and affect the production of antimicrobial peptides by intestinal epithelial cells^89^, but whether or not obesity alters the activity of enteric neurons is not clear.

### Microbial metabolites control immune system function during obesity

Just as the immune system exerts control on the intestinal microbiota, the microbiota also affects the composition and function of the immune system, in part through the production of microbial metabolites. SCFAs promote anti-inflammatory T cell responses by enhancing regulatory T cell function and IL-10 secretion^90,91^. SCFAs also metabolically reprogram B cells to support intestinal antibody responses, such as increasing numbers of IgA^+^ ASCs and IgA-coating of bacteria^92^. SCFAs polarize intestinal macrophages to an anti-inflammatory phenotype by increasing oxidative phosphorylation and lipid metabolism and enable mononuclear phagocytes to convert Vitamin A into retinoic acid, thereby promoting the synthesis of intestinal IgA^93,94^.

Other metabolites, with links to the microbiome, can also trigger GPR responses. For example, succinate can bind GPR91 on dendritic cells, triggering intracellular calcium flux and downstream immune responses^95^. Lactic acid and pyruvic acid can control the ability of mononuclear phagocytes to extend their dendrites and sample bacterial antigen through GPR31^96^. Imidazole propionate is a histidine-derived bacterial metabolite that can inhibit insulin signaling at the insulin receptor-substrate level during T2D^69^. Insulin receptor signaling can stimulate immune cells^97^; therefore, it would be interesting to see if such metabolites can alter immune cell-intrinsic insulin receptor signaling to affect disease outcomes.

AhR binds endogenous metabolites, dietary components, and microbiota products, and generally promotes the survival and function of intestinal immune cells^98^. Immune cell-specific activation of the AhR promotes Treg cell secretion of IL-10^99^, and ILC3 production of IL-22^100^, as well as regulating B cell class-switching and differentiation into ASCs^101^. A Western diet is associated with a reduction in intestinal microbial species that generate AhR ligands, which ultimately might lead to the reduced levels of IL-22 that occurs in diet-induced obesity, which can, in turn, reduce intestinal barrier function^63^. On the other hand, intestinal AhR signaling can also promote intestinal inflammation in some cases. Microbial produced oxazoles can induce AhR signaling on intestinal epithelial cells, which in turn limits CD1d-restricted production of IL-10, thereby activating iNKTs to contribute to intestinal inflammation via IL-13 and IFNγ production^102^. Indigo, an AhR ligand, which is chemically similar to tryptophan metabolites, can boost immunosuppressive and barrier protective immune responses to alleviate obesity-associated glucose dysregulation and insulin resistance in high-fat diet-fed mice^103^. However, the effect of AhR binding metabolites on intestinal immune cell function is probably dose-sensitive and thereby requires thorough dose-dependent assessment, as excessive AhR-induced Cyp1a1 production in the intestines can feedback to compromise levels of AhR ligands, resulting in smaller ILC3 and Th17 populations^104^.

Bile acid receptors FXR and G protein-coupled bile acid receptor 1 (TGR5) have the potential to regulate intestinal inflammation. Bile acid signaling through these receptors increases the anti-inflammatory profile of mononuclear phagocytes cells and T cells^105,106^. Modulation of intestine-specific FXR signaling via administration of chemical compounds seems to be ligand-specific. In one study, intestinally restricted FXR agonism with oral administration of fexaramine induced fibroblast growth factor 15 and enhanced thermogenesis^64^. Another study showed that administration of glycine-β-muricholic acid, which preferentially inhibits intestinal FXR, improved metabolic parameters, such as insulin resistance, glucose tolerance, and liver steatosis, by reducing intestinal ceramides^107^. Interestingly, bile acid metabolites have been shown to be essential for the maintenance of colonic RORγt^+^ Treg cells and have been linked with controlling intestinal inflammation in a DSS mouse model of colitis^108,109^. Given the broad effects of intestinal FXR on a variety of cells, understand its effect on the different immune populations within the intestine during obesity is important.

## Intestinal immunity in obesity-related therapy

The intestinal immune compartment and microbiota regulate metabolic disease outcomes in a manner that entails crosstalk with one another. Our understanding of this immune cell-microbiota axis has provided new insight into the modes of action of existing therapies as well as in identifying new therapeutic strategies, some of which are discussed in the next section.

### Clinical and lifestyle Interventions

The intestinal microbial-immune cell axis is affected by the administration of common therapeutic strategies to treat T2D, such as metformin or bariatric surgery, as well as lifestyle adjustments such as dietary modifications and exercise. Treatment with metformin remodels the microbiota by promoting the growth of glucose-sensitizing *Akkermansia muciniphilia* and inhibiting the growth of pathogenic *Bacteroides fragilis*, altering levels of bile acids such as glycoursodeoxycholic acid, and further skewing immune cells to an anti-inflammatory phenotype^110–112^. Bariatric surgery induces long-term changes in the intestinal microbiota and accompanies shifts in bile acid metabolism that are associated with increases in FXR signaling, as well as reductions in inflammation (primarily in VAT) via decreased T cell and macrophage accumulation and inflammatory function^113,114^. Interestingly, both metformin and bariatric surgery can increase levels of fecal secretory IgA, indicating that they both affect mucosal B cell responses^6^.

Alterations in diet, eating habits (such as time-restricted eating), and physical activity are at the forefront of lifestyle adjustments in the management of obesity-related insulin resistance. Some microbiota can metabolize polyunsaturated fatty acids to generate metabolites such as 10-hydroxy-*cis*-12-octadecenoic acid, which can attenuate insulin resistance and inflammation, through activation of GPR40 and GPR120, and GLP-1 secretion^115^. Administration of a diet high in dietary fibers to patients with T2D promoted the abundance of SCFA-producing microbes and alleviated disease-associated outcomes^116^, and in mice fed a Western diet, administration of the fiber inulin prevented disease-associated intestinal mucus penetrability and growth defects^117^. Whether the effects of SCFAs on mucus secretion are mediated by boosting intestinal Treg cell-derived IL-10 is not clear^118^.

Physiological interventions, such as exercise and caloric restriction, have metabolic benefits, some of which might be mediated by the intestinal microbiota and immune compartment. Exercise can increase the diversity of the microbiota, alter its composition, and attenuate intestinal inflammation^119,120^. Caloric restriction has also been shown to limit endotoxemia^121^, and decrease the number of circulating inflammatory monocytes^122^. Exercise and caloric restriction both have metformin mimicking effects on AMPK signaling and mapping these effects on intestinal immune cells might explain immunological changes within the intestines during such interventions.

### Probiotic, synbiotic, and synthetic therapies

A potential strategy to combat metabolic syndrome is the use of probiotic microbes, which are bacteria found in dietary supplements that are thought to have health benefits when consumed; however, the evidence surrounding the efficacy of this method in treating obesity-related insulin resistance is controversial, further complicated by variation in therapeutic effectiveness mediated by bacterial-strain specific effects^123^^.^ Nonetheless, as demonstrated by the administration of *A. muciniphila* to individuals with obesity, potentially insulin-sensitizing bacterial species might be administered safely to improve metabolic parameters^58^. As such, an exciting emerging avenue of work is to utilize unbiased computational screening methods to select microbes that are safe to colonize the host and favorably affect intestinal immune cell tolerogenic function and metabolic homeostasis^124^. Probiotic approaches can further be supplemented with prebiotics to enable the establishment of beneficial microbial species, a strategy known as “synbiotic therapy”. This method has been shown to be successful in reducing infant systemic inflammation in a randomized, double-blind, placebo-controlled trial to prevent sepsis and associated morbidity^125^.

Another interesting therapeutic avenue to pursue is the use of genetically engineered microbes. This approach has enabled the development of bacteria that secrete IL-10 to reduce intestinal inflammation^126^ and has been used as T1D and T2D therapy via administration of GLP1^127^ or *N*-acylphosphatidylethanolamine^128^ secreting synthetic bacteria, to improve glucose homeostasis.

Obesity is associated with compromised oral tolerance; orally fed antigens inhibit immune responses both systemically and locally in the intestines^2^. In a process that is dependent upon the microbiome, oral antigens are taken up by CX3CR1^+^ mononuclear phagocytes and transferred to CD103^+^ dendritic cells to induce anti-inflammatory T cell responses^129^. As such, the oral administration of beneficial microbes might promote mononuclear phagocyte function and induce intestinal Treg cell responses, enabling the reversal of inflammation in the lamina propria. Whether improved oral tolerance to pasteurized bacterial antigen is partly responsible for improvements in metabolic parameters in patients with obesity who are treated with pasteurized *A. muciniphila* is not clear.

Finally, an obesity-associated dysbiotic microbiota might be utilized to “vaccinate” individuals, as administration of the intestinal contents of obese mice has been shown to protect against obesity-associated insulin resistance, reduce meta-inflammation and restore the diminished numbers of intestinal Treg cells in mice fed a high-fat diet^130^. Similarly, immunization of high-fat diet-fed mice with flagellin, a protein highly expressed in some motile inflammatory microbes that can penetrate the mucus layer, decreases intestinal inflammation, increases the abundance of flagellin-specific IgA, and improves overall adiposity^131^. The precise mechanism responsible for beneficial outcomes of this vaccination strategy is unclear, but probably involves adaptive immune responses, such as via Tfh cells and IgA B cells and ASCs, to generate strong memory responses against pathogenic microbiota^130^. This vaccination approach also creates the possibility of selecting more specific pathogenic/obesogenic bacterial species or proteins for vaccination to prevent obesity-associated metabolic disease.

## Environmental and social factors

Personalized nutrition applies the idea that interindividual variations in intestinal microbiota can control differing metabolic responses to dietary intake^132^. This interindividual variability also affects the efficacy of probiotic therapy and the host microbiota is central to rendering an individual “resistant” or “permissive” to this approach^133^. Indeed, after taking antibiotics, probiotics can delay intestinal microbiome reconstitution, whereas autologous fecal matter transplant results in better restoration^134^.

Evidence indicates that the geography of the host can help to shape personal microbial communities, which can be further narrowed down to a neighborhood level, indicating that host genetics and culture are partly responsible^135,136^. In line with these observations, geographical migration has been reported to affect the human microbiome. Thai immigrants residing within the USA displayed a loss in native bacterial species and fiber degrading enzymes, which were associated with high body weight (BMI > 25) in immigrant communities^137^. These findings raise the idea of a corresponding inter-individual intestinal immune system linked to societal-induced changes in the microbiome.

Several factors can alter the intestinal microbiota during early infant life, such as mode of newborn delivery, use of formula feeding or exposure to antibiotics, which might have life-long ramifications for the adult microbiome and intestinal immune system^138,139^. C-section is associated with colonization of opportunistic bacteria, which could contribute to obesity and associated metabolic outcomes^140^. Supplying infants with prebiotic-enriched formula might induce SCFA promoting an environment that could protect against intestinal inflammation and obesity^141^. These ideas highlight the complexity of issues needed for efficient personalized nutrition.

## Working model

**Fig. 4:**
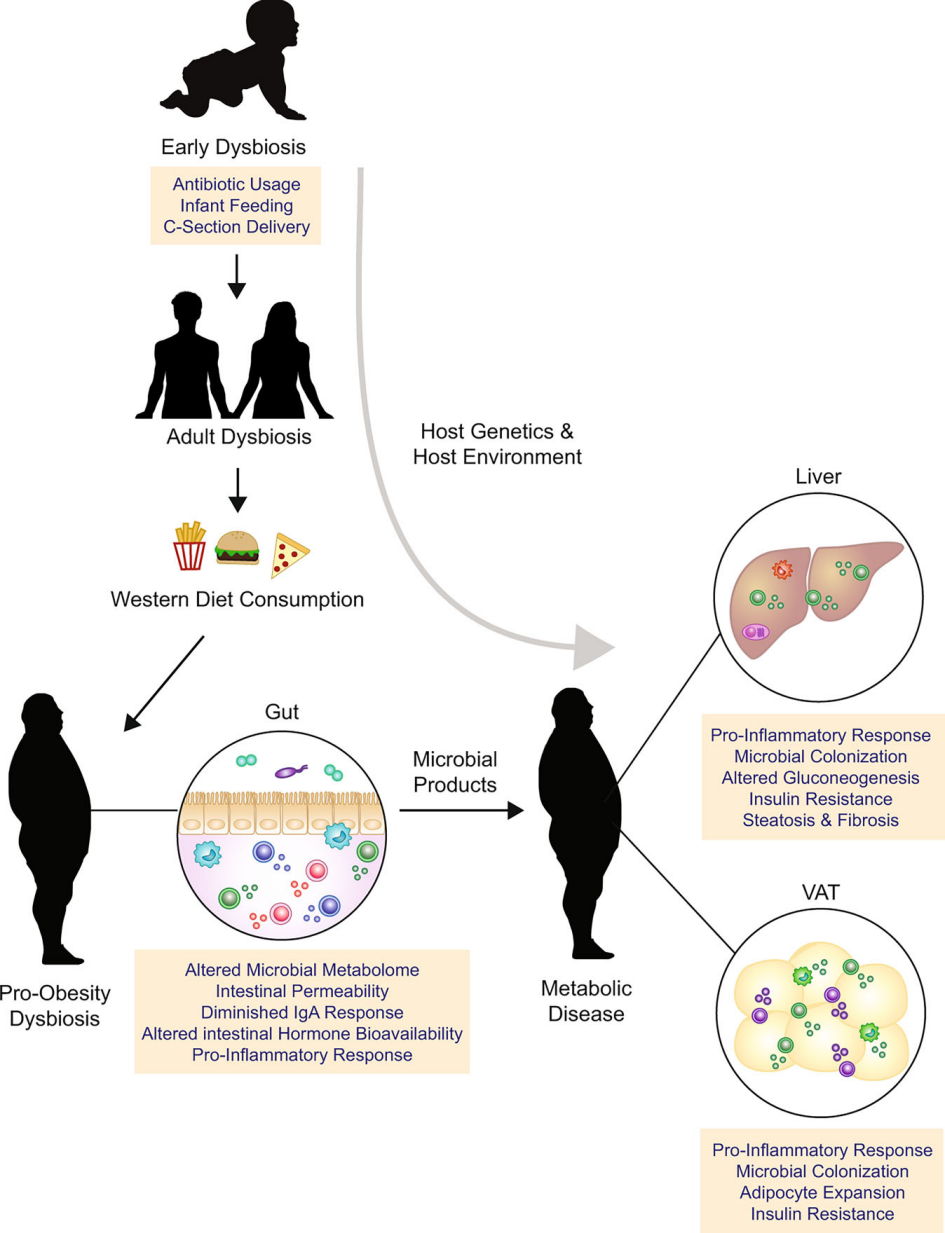
A working model of intestinal-driven metabolic disease. We propose that early life factors induce microbial dysbiosis, which can extend to adult life and potentiate the microbiome towards an obesogenic phenotype. Consumption of a Western diet results in a dietary trigger that further establishes pro-obesity intestinal microbial dysbiosis and a shift in the intestinal immune landscape towards a pro-inflammatory phenotype with limited intestinal IgA responses. Intestinal immune changes are also poised to alter intestinal hormone bioavailability. Low-grade inflammation coupled with dietary factors promotes intestinal permeability and facilitates the leakage of microbial ligands systemically. Consequently, penetration of microbial antigens and colonization of microbes at metabolic sites, such as the liver and visceral adipose tissue (VAT), affects tissue-specific metabolic processes and tissue-specific immune cell functionality. These processes are consistently affected by interactions between the host environment and genetics, ultimately resulting in the development of metabolic complications such as insulin resistance and non-alcoholic fatty liver disease.

We propose a multi-hit hypothesis that controls the crosstalk between the host microbiome and immune system in regulating obesity-associated metabolic outcomes (Fig. 4). Initially, an interface between the host microbiome and immune system is achieved at birth, where microorganisms from the environment establish symbiosis with newborns^138^. In humans, the first 3 months of life are a critical period for the stereotypic development of immune cells, which is perturbed in infants experiencing microbial dysbiosis^139^. Accordingly, germ-free mice have defects in the relative abundance of important intestinal immune cells required for mutualism (i.e., symbiosis) with intestinal commensal microbes, such as CD4^+^ T cells and IgA^+^ ASCs^142,143^. Early-life microbial dysbiosis as a consequence of the environmental variables discussed in this Review, such as host genetics, mode of delivery, host geography and antibiotic usage, potentially act as a trigger that affects intestinal immune cell function and potentially increases susceptibility to obesity-associated meta-inflammation.

Western diets that are rich in saturated fats, red meat, and sugars, while lacking in dietary fibers and vegetables, are a major risk factor that contributes to the development of obesity^144^. The consumption of a Western diet is the second key interaction that affects the host microbiota-immune cell axis and alters microbial composition from early infancy to adulthood. For example, diets rich in fats result in an over-representation of pathogenic LPS-expressing bacteria and subsequent metabolic endotoxemia^34^. The type of fat in the diet is also important. Compared with mice fed a diet high in unsaturated fats, mice provided with a diet high in saturated fats acquire a distinct intestinal microbiota composition that directly promotes pro-inflammatory processes via TLR-MyD88 signaling^145^. This change in fat intake might also affect the type of secondary bile acids produced by intestinal microbes. Similarly, excessive intake of dietary sugars, such as fructose and glucose, is associated with intestinal microbial dysbiosis and an increase in the abundance of opportunistic Proteobacteria at the phylum level^146,147^. Excessive intake of red meat results in increased microbial metabolism of dietary l-carnitine and trimethylalamine N-oxide production, which promote cholesterol accumulation and the pro-inflammatory output of macrophages^148^. Concurrently, removal of vegetables from the diet would diminish the supply of dietary fiber necessary for the production of SCFAs, herbal metabolites required for AhR activation, and Vitamin A essential for the generation of retinoic acid and IgA^149–151^. Therefore, the Western diet equips microbiota with the tools necessary to counter a tolerogenic immune system. These changes to the intestinal immune system also skew it away from reparative programs and have the capacity to disrupt the intestinal barrier^98^.

Third, hyperglycemia as a consequence of a Western diet facilitates intestinal epithelial cell reprogramming and reinforces a loss in intestinal barrier integrity, increasing penetration of immune stimulatory microbial ligands into the basal portion of intestinal epithelial cells, the lamina propria, and vasculature^152^. This leakage of microbial products further promotes a cascade of pro-inflammatory events in the local intestinal immune landscape, resulting in increases in pro-inflammatory cytokines, and decreases in tolerogenic anti-inflammatory cytokines and IgA antibodies. A shift toward pro-inflammatory cytokine production can disrupt tight junction proteins and negatively affect some commensal microbes, such as through the IFNγ–*Irgm1* axis^88^. Concurrently a loss in neutralizing IgA antibodies further facilitates an increased expansion of opportunistic microbes and associated penetration of pathogenic microbial products, thereby establishing a positive feedback loop^6,7^.

Drainage of microbial products and bacterial communities into metabolic tissues, such as the VAT and liver, results in reprogramming of tissue-specific inflammatory cells, associated with an expansion and recruitment of pro-inflammatory immune cells and a loss in tissue-resident tolerogenic immune cells^98^. In the VAT, these processes can potentially facilitate a loss in thermogenic properties, the formation of crown-like structures, and altered adipokine secretion^4^. In the liver, this process contributes to progression of NAFLD and transition to NASH, categorized by liver steatosis, hepatocyte degeneration, hepatic insulin resistance and the onset of hepatic fibrosis^3^. Though compensatory immune mechanisms at metabolic tissues are initiated, such as the emergence of Trem2^+^ macrophages, that regulate lipid homeostasis in adipose tissue^153^, and anti-inflammatory IgA ASCs that are possibly of intestinal origin^47^, we hypothesize that these mechanisms are probably not sufficient for preventing the development of obesity-related metabolic disease. Cumulatively, these effects can trigger a chronic systemic pro-inflammatory response and establish obesity-associated metabolic disease.

## Conclusion

Although major advances have been made in the field of obesity-associated intestinal immunometabolism, future work should continue building our understanding of the immune cell-microbiota axis, which fundamentally affects the outcomes associated with metabolic syndrome. Moving forward, it is essential to incorporate large scale analyses that better dissect these relations, such as imaging mass cytometry to visualize the immune landscape paired with single-cell RNA sequencing to obtain transcriptional profiles and facilitate the discovery of diet-induced metabolic changes to intestinal immune phenotypes, activation pathways, antigen receptor diversity, and even predict developmental lineages and cellular dynamics within the intestines of individuals who are obese.

Similarly, studies should further conduct unbiased large-scale omics analyses on the intestinal bacteria as well as their metabolome to identify important microbes and metabolites that can be targeted or boosted to ameliorate metabolic syndrome. The microbiota associated with obesity and the systemic metabolome should also be screened against specific receptors, as has been done for GPCRs under healthy steady-state conditions, which will identify diet–microbe–host axes that are altered between individuals who are obese versus lean^154^. Importantly, a consideration for experimental models and controls needs to be made when assessing a role for the intestinal microbiome in health and disease. When assessing mice with genetic differences, littermate breeding strategies should be incorporated to standardize the microbiota and limit incorrect conclusions^155^. Additionally, germ-free mice have perturbations in the generation and function of many immune cells, which confounds their use as tools to identify the causal role of microbes via fecal matter transplants or administration of enriched or purified bacterial communities. Nonetheless, assessing the microbial metabolome in inoculated germ-free recipients can yield mechanistic insights and provide a starting point for screening immune cell function in vivo against metabolites of interest. Furthermore, the intestinal microbiota includes an array of organisms other than bacteria, such as viruses, fungi, bacteriophages, and protists, which might be implicated in the progression of metabolic disease. Further investigation is needed to understand how these organisms might affect the bacterial microbiota and immune cells.

Understanding intestinal immune cell signaling axes and crosstalk with intestinal microbes is important for the identification of therapeutic targets to treat obesity and metabolic syndrome. With future research adding to our understanding of obesity-associated intestinal dysfunction and uncovering mechanisms of immune cell pathogenicity, we will be closer to the goal of developing safe therapies with improved effectiveness in obesity-related metabolic disease.

### Reporting summary

Further information on research design is available in the Nature Research Reporting Summary linked to this article.

## Supplementary information

Reporting Summary
